# Patient-Centered Risk Prediction, Prevention, and Intervention Platform (TIMELY) to Support the Continuum of Care in Coronary Artery Disease Using eHealth and Artificial Intelligence: Protocol for a Randomized Controlled Trial

**DOI:** 10.2196/66283

**Published:** 2025-08-14

**Authors:** Mirela Habibovic, Emma Douma, Hendrik Schäfer, Manuela Sestayo-Fernandez, Tom Roovers, Xin Sun, Henrik Schmidt, Mona Kotewitsch, Jos Widdershoven, David Cantarero-Prieto, Frank Mooren, Carlos Pena-Gil, José Rámon González Juanatey, Martin Schmidt, Hagen Malberg, Vassilis Tsakanikas, Dimitrios Fotiadis, Dimitris Gatsios, Jos Bosch, Willem Johan Kop, Boris Schmitz

**Affiliations:** 1 Department of Medical and Clinical Psychology Tilburg University Tilburg The Netherlands; 2 Department of Rehabilitation Sciences University of Witte / Herdecke Witten Germany; 3 Center for Medical Rehabilitation Clinic Königsfeld Ennepetal Germany; 4 Cardiology and Coronary Care Department Universidade de Santiago de Compostela Santiago de Compostela Spain; 5 Faculty of Social and Behavioural Sciences University of Amsterdam Amsterdam The Netherlands; 6 SEMDATEX Berlin Germany; 7 Department of Cardiology Elisabeth-TweeSteden Ziekenhuis Tilburg The Netherlands; 8 Instituto de Investigación Marqués de Valdecilla Cantabria Spain; 9 Faculty of Electrical and Computer Engineering Technische Universität Dresden Dresden Germany; 10 Foundation for Research and Technology Hellas Ioannina Greece; 11 Department of Materials Science and Engineering University of Ioannina Ioannina Greece; 12 Capamed Ioannina Greece

**Keywords:** cardiovascular disease, cardiac rehabilitation, eHealth, lifestyle, physical activity, artificial intelligence, AI

## Abstract

**Background:**

Cardiac rehabilitation (CR) programs have shown promising results in tertiary prevention. However, achieving sustained behavioral changes remains a challenge.

**Objective:**

The TIMELY project aims to develop and evaluate a personalized, artificial intelligence–driven eHealth platform and app to support sustainable behavior change during phase-3 CR, subsequently reducing cardiac risk scores.

**Methods:**

An international, multicenter, randomized controlled trial will be conducted to evaluate the effectiveness of the TIMELY integrated platform and app. A total of 360 patients with cardiac conditions will be approached to participate in the study across Germany, Spain, and the Netherlands. Patients will be randomly assigned (1:1) to either the intervention group or the control group (care as usual). The intervention group will receive fully web-based, behavior change support through the TIMELY app, with personalized exercise prescription, chatbot assistance, and monitoring devices (eg, activity watch). Both groups will continue to receive care as usual, complete validated questionnaires, undergo physical tests, and provide blood samples. Assessments will be conducted at baseline and at 3, 6, and 12 months.

**Results:**

Trial recruitment has been finalized in July 2024. A total of 358 patients have been recruited and randomly assigned to the intervention (n=180, 50.1%) or care-as-usual (n=178, 49.7%) condition. As of January 2025, the 6-month follow-up has been completed for all participants. Follow-up data collection will be completed in May 2025. Results regarding the primary and secondary objectives are expected in September 2025.

**Conclusions:**

This project will test a personalized platform and app, supported by artificial intelligence and designed to support health behavior change during phase-3 CR. It will target multiple health behaviors, with a primary focus on physical activity and fitness levels, using an integrated approach.

**Trial Registration:**

ClinicalTrials.gov NCT05955625; https://clinicaltrials.gov/ct2/show/NCT05955625

**International Registered Report Identifier (IRRID):**

DERR1-10.2196/66283

## Introduction

### Background

Cardiovascular diseases are the leading cause of death worldwide, accounting for approximately 17 million deaths annually [1]. Improvements in cardiac treatment over the past decades have increased the number of people surviving cardiac events and living with cardiac disease. Consequently, cardiac disease management and tertiary prevention of adverse outcomes such as recurrent cardiac events or mortality have become increasingly important in clinical practice and patients’ lives [2]. Cardiac rehabilitation (CR) programs play an important role in this risk factor management and have been demonstrated to improve patient outcomes effectively [3].

### Cardiac Rehabilitation

The World Health Organization defines CR as “the sum of activities required to influence favorably the underlying cause of the disease, as well as to provide the best possible physical, mental, and social conditions, so that the patients may, by their own efforts, preserve or resume when lost as normal a place as possible in the community” [4]. CR is a guideline-recommended [5], multidisciplinary, multimodal tertiary prevention program consisting of modules that include exercise training, optimization of medical treatment and risk factor management, smoking cessation, heart-healthy dietary habits, risk factor education, and psychosocial counseling [6,7]. Phase 1 is the inpatient care that is characterized by the implementation of modern acute coronary revascularization techniques as well as the application of effective acute and long-term pharmacotherapy aiming at early mobilization of the patient after a cardiac event or surgery. This phase is followed by phase-2 CR, which typically addresses patient’s personal risk factor modification by adopting a healthy lifestyle. This represents the most important challenge, often described as the intention-behavior gap. Changes achieved during phase 2 are aimed at being consolidated in phase-3 CR. Long-term maintenance of health behavior changes after CR (ie, during phase-3 CR) varies substantially in the level of support offered to patients [8]. In most countries, the availability of phase-3 CR support is limited because of reimbursement policies. When center-based support is offered, only a small subgroup of patients attends these sessions, often due to a lack of personalized guidance and logistical challenges such as time constraints and travel burden [9,10]. These patient- and health system–related barriers contribute to low adherence to and uptake of long-term rehabilitation and cardiovascular health maintenance programs despite their potential health benefits [11,12]. Therefore, it is of utmost importance to explore delivery modes for long-term CR maintenance that better align with the needs of both patients and healthcare providers, while addressing existing barriers.

### eHealth in CR

Web-based care modalities for CR might be the way forward to address the challenges associated with “traditional” in-person CR. These modalities offer the opportunity to provide *personalized care* or support to patients at their convenience, while also being *scalable* and *cost-effective* [13-15]. Such an approach should include both a continuous risk analysis by monitoring multiple disease-related prognostic parameters as well as regular support and guidance for a cardiac healthy lifestyle. A recent meta-analysis by Heimer et al [16] demonstrated that eHealth-based, phase-3 CR is effective in increasing exercise capacity, improving quality of life, and decreasing blood pressure (BP). Another meta-analysis found that remote CR is associated with better behavioral, psychosocial, and health service use outcomes [13,17]. These findings suggest that technological innovations can help develop a continuum of care after initial center-based CR to sustain its benefits and improve health outcomes. Therefore, recent studies have focused on the development of behavioral interventions to support long-term CR maintenance [18-20].

### Cardiac Healthy Behavior Change

Developing and maintaining a cardiac-healthy lifestyle is of utmost importance to improve prognosis and reduce mortality risk in people with cardiac disease. It has been demonstrated by multiple studies [21,22] and advocated by others [23,24] that health behaviors, and in particular physical activity, should be addressed to improve cardiac health. Changing health behavior is a challenging endeavor, and only few people succeed [25]. Particularly, patients who face complicated disease regimen or adjustment problems (psychological or psychosocial) due to their illness (such as coronary artery disease [CAD]) may experience health behavior change as burdensome. This is also reflected by the typically temporary nature of the changes in health behavior that do not persist in the long term [26]. To promote sustainable behavior change, the application of behavior change techniques (BCTs) has been advocated [27]. The current literature indicates that a variety of BCTs may be implemented in CR programs to enhance their effectiveness. The TIMELY consortium conducted 2 studies that guided the development of the TIMELY intervention, 1 focusing on the patient perspective (qualitative) [28] and 1 evaluating existing literature through a systematic review [29]. Triangulating the findings from the studies revealed that specific BCTs can be successfully implemented to improve health behavior and clinical outcomes when they align with patients’ preferences and are optimally matched with target behaviors.

### TIMELY

**Figure 1:**
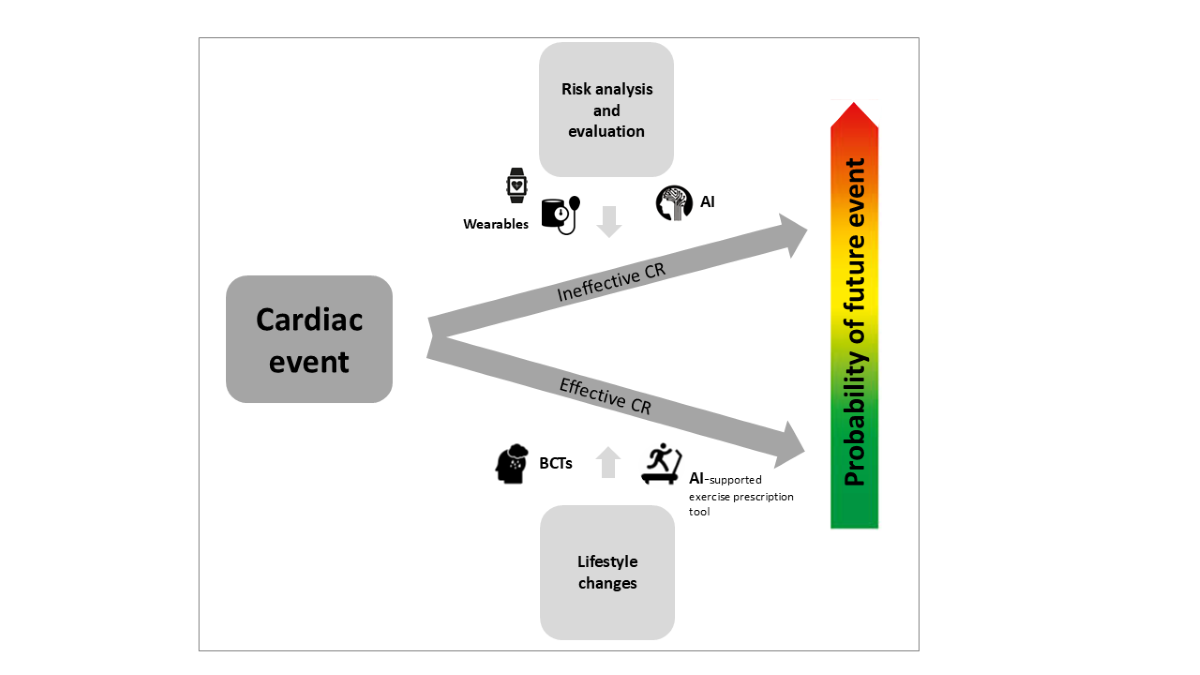
The TIMELY approach. AI: artificial intelligence; BCT: behavior change technique; CR: cardiac rehabilitation.

To overcome current barriers related to phase-3 CR programs, the TIMELY trial aims to evaluate the cost-effectiveness of a personalized eHealth platform and health behavior change app for patients with CAD. This platform and app will provide support for health behavior change (eg, artificial intelligence [AI]–supported personalized exercise prescription [EP]) and facilitate disease-related self-management based on current clinical guidelines and in accordance with patients’ individual needs. The TIMELY intervention will integrate (through the TIMELY platform) demographic, clinical, and psychosocial data to assess cardiac risk and support patients in maintaining and improving health behaviors via app-based feedback and prompts. Therefore, the TIMELY intervention is designed to preserve the benefits of CR and reduce cardiac risk in patients with CAD. At the same time, this approach reduces the burden on health care providers and the health care system. The TIMELY intervention is the first intervention for patients with CAD to support its users with physical activity and stress management through a chatbot function, and to offer a personalized weekly EP that is adapted based on exercise parameters collected throughout the week [30]. Previous interventions using increasing exercise goals based the goals on single parameters such as baseline activity levels or heart rate reserve, whereas the EP in the TIMELY intervention is based on several medical, demographic, and behavioral parameters [31,32]. Figure 1 shows a graphic outline of the TIMELY approach. It is hypothesized that participants who follow the TIMELY intervention, as compared to care as usual, will have a lower 10-year cardiac risk mortality and improved physical fitness over time. In addition, they are expected to have better physical and mental well-being and quality of life.

## Methods

### Design

The TIMELY study is an international (Germany, Spain, and the Netherlands), multicenter, randomized controlled trial. Patients randomly assigned to the intervention condition will receive a 6-month, app-based, personalized behavior change intervention. Assessments will be performed at baseline and at 3, 6, and 12 months.

### Ethical Considerations

The TIMELY trial has been registered at ClinicalTrials.gov (NCT05955625) and has been approved by the Medical Ethics Committee (METC-Brabant NL82723.028.22/P2251 Tilburg, protocol V9 24-05-2023, the Netherlands). Local approvals from the participating centers (Germany: Z-125/2023; Spain: CEIm-G 2023/119) have been obtained. Data collection was initiated after receiving a signed informed consent from the participants. Participants have the option to opt out at any time—before, during, or after data collection—in which case their data will be deleted. Data will be pseudonymized, and only the research team from each center will have access to the key list for their center. A data sharing agreement between the participating centers has been signed for sharing pseudonymized data for data analysis purposes. Informed consent covers secondary analysis without additional consent. Participants were not reimbursed for participation except for study-related expenses (eg, parking costs).

### Monitoring

Tilburg University has been appointed as the trial sponsor and will perform regular monitoring of the trial. This includes 2 visits (midterm and closing-out) to the clinical centers in Germany and Spain. During this visit, the investigator site file will be reviewed, and random checks of 20% of the participants regarding clinical data and questionnaires will be performed. Informed consent forms will be checked for all participants. In the Netherlands, the monitoring visits will be performed by an independent researcher from Tilburg University who is not affiliated with the TIMELY trial.

Throughout the study period, monitoring of serious adverse events will be performed per center. If serious adverse events are reported by patients or discovered in the patient record, this will be reported to the sponsor, who will register the events within 1 week at the Dutch Central Committee on Research Involving Human Subjects. The events will also be registered at the German Federal Institute for Drugs and Medical Devices.

### Dissemination Policy

The results of the TIMELY trial will be disseminated in academic and health care settings through scientific publications, symposia, and conference visits. Study participants will be informed about the results via a newsletter containing the highlights of the study outcomes. To reach a broader audience, local newspapers will be contacted to disseminate the results among the general public.

### Study Population

Women and men diagnosed with CAD will be included in the study. The following eligibility criteria have been defined:

- Inclusion criteria: (1) aged ≥18 years (there is no a priori upper age limit); (2) documented stable CAD and referred for CR (at >2 weeks but <10 weeks after percutaneous coronary intervention or >4 weeks but <12 weeks after coronary artery bypass grafting or myocardial infarction: with or without ST-segment elevation), or having documented CAD by coronary angiography (stenosis in a major coronary artery >50%); (3) access and ability to operate a smartphone; and (4) proficiency in the native language of the country (ie, German, Spanish, and Dutch).
- Exclusion criteria: (1) unable to fully understand the provided study information and consequences of participating in the study; (2) presence of an impairment interfering with the use of the app or devices (eg, blindness and wheelchair bound); (3) known diagnosis of an active malignant tumor (cancer) or any other medical condition associated with a life expectancy of <1 year; (4) cardiovascular, cerebrovascular, or other unstable medical condition; (5) having a pacemaker or life vest; and (6) refusal to provide informed consent. If any of the exclusion criteria emerge during the 1-year duration of the project, participants will be withdrawn from the study.

### Sample Size

A total of 360 patients will be enrolled across 3 participating centers: Hospital Universitario de Santiago de Compostela, Spain (120/360, 33.3%); Clinic Königsfeld of the German Pension Fund at the University of Witten/Herdecke, Ennepetal, Germany (120/360, 33.3%); and Elisabeth-TweeSteden Hospital, Tilburg, the Netherlands (120/360, 33.3%). At each center, patients will be randomly assigned, with 60 (50%) out of 120 receiving the intervention in addition to care as usual, and 60 (50%) receiving care as usual. Of 360 patients, 180 (50%) will receive the intervention, and 180 (50%) patients will receive care as usual.

On the basis of participation rates in previous randomized controlled trials [33], it is expected that approximately 33% of patients will decline participation. It is expected that a total of 480 patients will have to be approached to include 360 participants across the 3 study sites. Sample size calculations were based on medium-large effect sizes and statistical power analyses were conducted using G*Power (version 3.1.9.4; Heinrich-Heine-Universität Düsseldorf) [34]. A sample of 120 patients per center will enable detection of a medium effect size. The *f*^2^ for the between-within group interaction is 0.15 in a mixed model ANOVA, with a power of 0.80 and a two-sided α level of 0.025 (0.05/2, as two primary outcomes will be evaluated; details are provided in the *Study Objective* section). In brief, the set of variables for this analysis was based on a 2 (between-group: intervention vs control)×2 (repeated measures at baseline and 6 months) mixed model ANOVA, assuming sphericity and a correlation of 0.3 between the repeated measures.

### Randomization and Blinding

Participants will be randomly assigned (1:1) to either the intervention group or the care-as-usual group. A computer-generated randomization sequence will be delivered, in an Excel (Microsoft Corp) file, to each recruitment site and will be concealed by one of the researchers by covering the allocation numbers with “black paint.” Randomization will be conducted and dated, per patient, by the researcher after the participant has signed the informed consent and baseline assessments have been performed.

Given the nature of the study, blinding of the researchers or participants during the behavioral intervention trial is not possible. However, the analysis of blood samples and all other outcome measures will be conducted by research team members who are blinded to the participants’ group assignment.

### Study Procedure

Approximately 2 weeks before completing CR, patients who have started the program will be approached by a research assistant for participation after eligibility (inclusion and exclusion criteria) has been checked by the local primary researcher or clinician. Patients who are eligible for CR but decide not to take part in the program will be approached after the research assistant is notified of patients’ participation status. If a patient expresses interest in participating, study information will be provided both verbally and in writing (patient information letter including the informed consent form).

For patients who consent to participate in the study, the baseline assessment includes physical exercise and functional tests, body composition analysis, blood draws (Multimedia Appendix 1), and completion of validated (web-based) questionnaires. Clinical data will be provided by the patient through an inquiry of selected data from the patient’s medical record. At 3 months, all participants will receive an online link to fill in a set of validated questionnaires to evaluate potential early changes that occur during the intervention. At 6 months, participants will be invited to the hospital to return the study devices (refer to the *Study Procedure* section) and undergo the second physical exercise testing, blood sampling, and questionnaire assessment (online). At 12 months, patients will be invited for the final hospital visit during which the same assessments as obtained at the 6-month follow-up will be performed. For patients who do not fill in the questionnaires within a given period of 10 days, up to 3 reminder phone calls will be made by the researcher.

### Study Objectives

#### Overview

The overall aim of the TIMELY study is to reduce the cardiac risk score through personalized health behavior change. The main focus is on the improvement of physical activity levels. All components of the TIMELY program should result in a reduction in the risk of recurrent cardiac events. All primary and secondary outcomes will be obtained at baseline; 6 months (the main outcome time point); and 12 months (long-term follow-up).

#### Primary Objectives

The primary objective of the TIMELY study is to investigate whether the TIMELY intervention is superior to usual care in terms of the following:

- Reducing the risk of 10-year mortality assessed by a validated biomarker risk score (CoroPredict, primary biomedical outcome) from baseline to 6 months. The Coropredict score assesses cardiovascular risk using a combination of laboratory-based parameters (including hemoglobin A_1c_, N-terminal pro-B-type natriuretic peptide, high-sensitivity troponin I, cystatin C, and high-sensitivity C-reactive protein) and demographic information (age, sex, and smoking status) [35].
- Increasing the 6-minute walking test distance (primary behavioral outcome indicating functional fitness level) from baseline to 6 months.

#### Secondary Outcomes and Secondary Objectives

Improvements in the primary outcomes are expected to be associated with improvements in the following secondary outcomes:

- Increase in physical activity: physical activity levels during daily life will be assessed using the International Physical Activity Questionnaire [36].
- Increase in cardiovascular exercise tolerance: measures of cardiovascular responses to exercise will be based on graded symptom-limited exercise tests, using maximum watt (watt/kg body weight) as the outcome.
- Healthy dietary habits: dietary habits will be assessed using the dietary questions from the validated Health Promoting Lifestyle Profiles-II questionnaire [37].
- Decrease in body weight: body weight will be measured in kilograms.
- Smoking cessation: smoking cessation will be assessed by the validated Fagerström questionnaire [38].
- Better medication adherence: medication adherence will be assessed using the validated Medication Adherence Report Scale questionnaire [39].
- Decrease in psychological stress levels: changes in psychological stress levels will be assessed using the Perceived Stress Scale [40].

The secondary objectives of the TIMELY project will be assessed using questionnaire data obtained at baseline and at 3, 6, and 12 months:

- To investigate whether the TIMELY intervention is superior to care as usual in terms of improvement in physical and mental well-being and quality of life.
- To investigate the feasibility and usability of the TIMELY intervention.
- To investigate whether the TIMELY intervention is superior to care as usual in terms of cost-effectiveness.

### TIMELY Intervention

#### Development

The TIMELY intervention, including all technical components (dashboard, app, and monitoring devices), was co-designed with input from patients and health care professionals (eg, cardiologists, cardiovascular nurses, and therapists). A living laboratory approach was followed to collect detailed insights into patients’ needs and expectations on functionalities and app design. For a detailed description of the input provided by patients, refer to Douma et al [28] and Schmitz et al [41]. Furthermore, an extensive systematic review was conducted [29], which indicated that self-monitoring of behavior, prompts or cues, instructions on how to perform the behavior, goal setting, and social support were most likely to result in change in physical activity. In addition, the findings showed that providing prompts or cues as part of behavior change was unlikely to stimulate smoking cessation in patients with CAD and the intervention was adapted accordingly. The BCTs that were identified from focus groups and the systematic review were included in the TIMELY app throughout the functionalities of the app (chatbot, information provision, and monitoring).

#### Monitoring of Physical Activity, Hemodynamics, and Electrocardiogram During Daily Life Activities

All patients randomly assigned to the intervention group will be equipped with an activity tracker (Vivosmart 4, Garmin), an upper-arm BP monitor (Tel-O-Graph BT, IEM), and a 3-channel Holter monitor (netECG, livetec). Data from the activity tracker will be collected via Bluetooth on the patients’ smartphone and transmitted directly to the TIMELY-Net server (Semdatex) using an application programming interface provided by FitRockr to comply with European data protection regulations. The BP monitor will be placed in the patient’s home, together with a data transmission hub (eConnect, IEM), which allows secured mobile transmission of data (GSM standard) via the IEM gateway to the TIMELY-Net server. Collected data are then displayed in the TIMELY dashboard for case managers and made available to patients via the TIMELY app on their mobile phones, which can be downloaded from the app store for Android and iOS devices (see below for a detailed description of the TIMELY app).

Electrocardiogram (ECG) monitor data will be analyzed retrospectively after the trial. In brief, data will be downloaded from the ECG device at each participating center and uploaded to the TIMELY platform. The data will then be analyzed by ECG specialists at the participating partner institution, TU Dresden, using deep learning technology as described elsewhere [42]. Clinicians at the 3 centers will be involved to verify incidental findings of ECG abnormalities.

#### TIMELY Platform and Infrastructure

**Figure 2:**
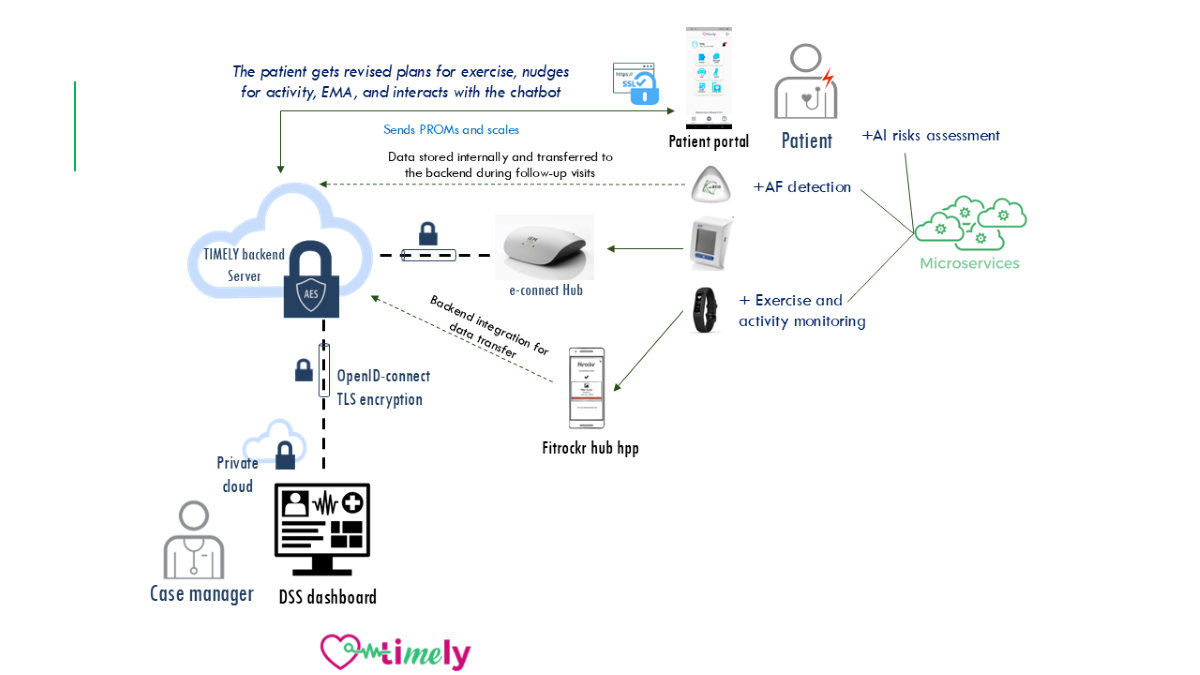
TIMELY-Net infrastructure. AES: Advanced Encryption Standard; AF: atrial fibrillation; AI: artificial intelligence; DSS: decision support system; EMA: ecological momentary assessment; PROM: patient-reported outcome measure; SSL: Secure Sockets Layer; TLS: Transport Layer Security.

Data obtained from the monitoring devices and via the TIMELY app (patient-reported outcome measures [PROMs]) will be processed on the integrative TIMELY platform (Figure 2). The platform allows the assignment of devices to registered patients and provides insights into patients’ functioning and progress to designated case managers. The platform was designed to ensure that no personally identifiable data are transmitted over the internet. Specifically, services are executed on the same server cluster to significantly reduce the number of off-site accessible application programming interface end points. These end points are protected by up-to-date authentication methods (OpenID-Connect) and are encrypted in transit using Transport Layer Security. Personal identifiable data are also encrypted in the database. The clinician dashboard is implemented as a separate plug-in in a certified medical office software (inSuite, Doc Cirrus) hosted on a private cloud (which is a type of cloud computing environment that is dedicated to a single organization that offers CR with TIMELY) in the same local network as the other services of the TIMELY-Net platform. The platform follows a microservice approach to implement different services and functionalities, including the following:

- Information from and to patients via the TIMELY mobile app (including messaging)
- Clinicians’ dashboard enabling case managers to assess structured patient data, definition of thresholds, and connected tasks or alerts as well as decision support systems (DSSs)
- Service to analyze 24-hour ECG data and detect cardiac arrhythmias such as atrial fibrillation (atrial fibrillation–detection)
- A chatbot to guide behavioral change
- Ecological momentary assessment service for assessment of PROMs via the mobile app
- EP DSS
- Risk prediction service to predict 10-year mortality risk in cardiovascular diseases (described in detail elsewhere) [43]

#### AI and Machine Learning

Demographic patient information and clinical data, including the ECG and exercise capacity results from the preceding clinic-based rehabilitation sessions, are entered into the risk profile database that is subsequently integrated and accessible to care providers and case managers via the TIMELY platform. Using machine learning–based algorithms, an optimal personalized exercise schedule is then developed and shared with the participants via the app (see below for details about the app). These exercise schedules are also adapted to the participant’s (changes in) actual activity levels over the past week to personalize the patient’s exercise levels during daily life activities.

AI is a key component of the TIMELY project as AI will support the modular, collaborative eHealth platform. AI is primarily directed toward the prediction of cardiac risk and complications. In addition, the targeted behavioral change interventions are designed to improve patients’ self-care and empowerment. AI will be primarily used to optimize risk prediction based on patients’ clinical and demographic background factors that will be integrated with the participants’ responses to the TIMELY eHealth intervention. The chatbot-based patient prompts will use preset and validated phrases, but not directly involve AI-based text, as this would create unpredictability of conversation content, which might be undesirable in patients with CAD. Thus, TIMELY involves a combination of AI-based risk prediction with patient-tailored personalized behavioral prompts via the chatbot. This approach will optimize long-term clinical care and secondary CAD prevention.

#### Clinical Dashboard

Information provided in the dashboard includes systolic and diastolic BP, heart rate, step count, floors climbed, calories burned, physical activities (type of activity, duration, and activity calories), sleep duration, and stress levels. Data can be displayed per day or as an average per week, or month. By setting alert thresholds on critical values such as missing data (ie, no data transmitted within 7 days), reported symptoms, or detected BP deviations, tasks are automatically generated with an indication of severity: high, medium, and low. These tasks help guide case managers in informing patients about changes in their health status that may require attention.

#### TIMELY App

**Figure 3:**
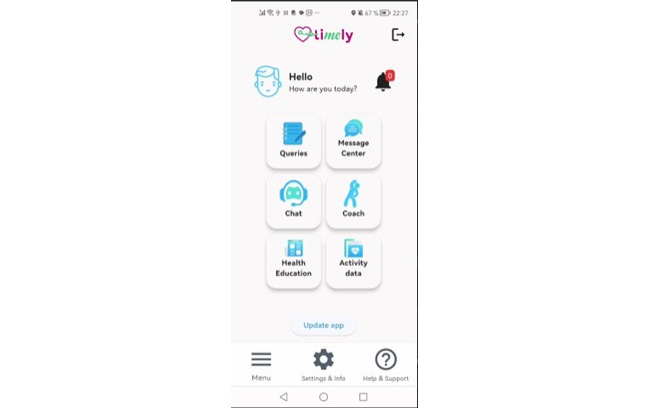
TIMELY app functionalities (screenshot).

The TIMELY app (available for Android and IOS devices) was developed following a simplistic design approach and serves two central purposes: (1) collecting self-report data or PROMs and (2) providing personalized information (from wearable or monitoring data) and advice regarding health behavior change (Figure 3). To achieve these goals, the app provides seven functionalities: (1) messaging function for nudging and information; (2) ecological momentary assessment diary assessment of symptoms, perceived stress, sleep quality, affective states, social behavior, motivation, and goal setting; (3) lifestyle and health information; (4) chatbot interaction guided by specific counseling techniques and methods; (5) weekly EP; (6) documentation and visualization of physical activity and vital signs; and (7) documentation and visualization of BP. The app was freely available for study participants and could be used by participants at their own time and pace.

The functionalities provided by the app (in combination with the microservices run on TIMELY-Net) are detailed in Textbox 1.

**Functionalities of the TIMELY app.**

- *Messaging function (“message center”)*: Patients can receive messages (push notifications), which are either sent manually by the case managers (technical feedback on the system, available updates, and instructions) or automatically generated messages. Automatic messages include information on available queries, feedback on achieved goals, and activity nudges. The activity nudges have a reminder function combined with health information and are generated from a large list of different messages to avoid redundancies. Nudging is integrated with weather information to suggest activity options—for example, “It's warm and sunny today, perfect for exercising outside”—and to help overcome barriers, such as “It's rainy today, but you can still enjoy a healthy indoor workout.” Moreover, geolocation nudging is used (based on GPS) to inform about the option to be physically active (“you are near a park, a good opportunity for a walk”).
- *Self-report assessments (“queries”) using ecological momentary assessment (EMA) diaries*: EMA (ie, brief diary-like questionnaires or “queries”) will be used for daily assessments of psychological and physical functioning and goal setting. This feature includes behavior change techniques (BCTs) such as self-monitoring of symptoms, perceived stress, sleep quality, mood states, health-related behaviors, and prompts or cues to provide responses.
- *Lifestyle and health information (“health education”)*: The app provides guideline-based information regarding cardiac health behaviors, benefits of regular physical exercise, and instructions on how to perform exercise (pictures and sample videos), medication, heart-healthy diet, stress management, smoking cessation, and risk factors selected by health care professionals (cardiologists, psychologists, and physical exercise specialists) using simple language. This section also provides web links to additional information provided by different societies and patient organizations. Several BCTs are part of this functionality, including *instructions on how to perform the behavior, information about health consequences, and demonstration of behaviors.*
- *Chatbot interaction (“chat”)*: The TIMELY app includes a conversational agent that uses user context and artificial intelligence (AI)–based cardiovascular disease risk prediction information, referred to here as the “chatbot.” Suggestions from the chatbot are based on domain knowledge of behavioral intervention theories and related BCTs [44]. The chatbot primarily focuses on improving physical activity levels and overcoming potential barriers such as fatigue, psychological stress, or the patient’s environmental setting that interferes with or facilitates physical activity [45]. The chatbot also assists patients with planning health behaviors and identifying unhelpful behaviors and cognitions, including training modules to mitigate them based on cognitive behavioral interventions and motivational interviewing.
- *Exercise prescription (EP; “coach”)*: During the intervention phase, patients will receive a personalized, guideline-based EP with weekly updates. The EP is generated automatically using a decision support system (DSS) for case managers developed as part of the TIMELY project. The design, functionality, and validation of the tool will be described in detail elsewhere. In brief, the rule-based tool was developed in accordance with the European Society of Cardiology Guidelines on EP [46] and follows the frequency, intensity, time, and type of exercise approach. It is composed of 2 integral functionalities: the permissibility report and EP. The patient’s medical history and current exercise-related status inform the EP module, and information on sports to avoid or suitable sports disciplines and cautionary information to minimize the risk of injury or exercise-related cardiovascular problems are provided. The EP functionality suggests an initial exercise program and weekly progressions, relying on an algorithm crafted from best practices found in the clinical guidelines for exercise progression [47]. For initial exercise programs, the EP considers the fitness level of the patient after the phase-2 cardiac rehabilitation (CR), while for progressions, it factors success in achieving the caloric goals (metabolic equivalents, determined using the activity tracker based on heart rate) established in the previous week’s program and any reported symptoms. This approach ensures that the exercise regimen remains tailored to the individual’s needs and goals, promoting personalized exercise recommendations. Performed exercise sessions with duration and activity calories (as percent of the weekly goal) are provided. The EP incorporates the BCT of *action planning, instructions on how to perform the behavior, and biofeedback.*
- *Goal setting and physical activity monitoring (“activity data”)*: Goal setting is a shared decision between the patient and case managers at the start. During the intervention, new goals are set by the case manager after receiving input from DSS. Patients are instructed to wear the activity tracker daily for 24 hours and wearing time (in percent) will be used (among other criteria) to define adherence to the program. Data on steps taken, physical exercise sessions performed (type, duration, activity calories, and percent of caloric goal), caloric expenditure, floors climbed, and heart rate are provided per day using simple, noncomplicated graphs. Data from previous weeks are provided for comparison and progress indication. For steps, a daily step goal is indicated in the graph. The step goal is discussed and defined with the patient during onboarding. Patients are informed about achievements via automated messaging weekly and can adjust the step goal if indicated using the diary function. This section of the app also provides feedback on set goals (eg, daily step goal) and goal achievement. The BCTs that are included in this feature are *biofeedback and feedback on outcomes of behavior.*
- *Blood pressure (BP)*: Patients can review their current and past BP data via the app and will be instructed to use the BP monitor at the same time every day. Similar to physical activity monitoring, feedback on BP data is incorporated into this feature.

The chatbot uses predefined scripts that have been previously validated [45] and translated into German, Dutch, and Spanish by native speakers.

In addition, a support function providing technical help and troubleshooting, including written instructions and videos, is available in the app.

#### The Role of the TIMELY Case Manager

In alignment with a realistic health care setting, case managers (rehabilitation nurses and health care assistants) will provide technological and app-specific support during the study. During the inclusion period, patients are onboarded by the case manager, which includes giving access to the app and related monitoring devices and ensuring that participants understand the study requirements and all provided functionalities. The case manager sets the first weekly step goal together with the patient, and the patient agrees to the first EP (shared decision-making). Within the first week after inclusion, the case manager will contact the participants to verify proper installation of the app and monitoring devices, ensuring data collection integrity. Participants will have the opportunity to ask questions about the device functionality. During the intervention period (6-month follow-up), patients will be contacted monthly to ensure intervention delivery and resolve any unforeseen technical difficulties. These follow-up calls are strictly for technical support and continued engagement, with no behavior change support provided. The case manager’s actions are guided by a comprehensive handbook that includes protocols for missing data transitions and threshold violations.

For safety reasons, the case manager reviews new EPs generated by the EP DSS every Monday, before the EP is sent to the patient’s phone. The DSS has been developed to assist case managers in their daily work, primarily focusing on the identification of patients with deviating variables who require further assistance. The EP tool was designed to prescribe exercise to patients based on the medical guidelines, individual clinical profile, and personal needs. The tool allows for weekly adjustments based on the patient’s progress. EP additionally requires information on the previous (past week) exercise program, feedback on program execution (including metabolic equivalents achieved and reported symptoms), and patient preferences (if any) on which exercise parameters to increase or decrease (endurance or resistance, intensity, number of sets, and frequency). On the basis of these parameters, a prescription is produced. This DSS helps case managers in prescribing safe, well-informed, and patient-centered exercises. Depending on the task category, case managers can use various modes to contact patients. This includes sending (prewritten) messages via the app to guarantee data availability (“please wear your activity tracker regularly”) or calling the patient for safety reasons in case of an incidental finding (eg, BP deviations less than or greater than preset limits). Patients can also request a phone call with the case manager if they experience any technical problems or have questions about the app or monitoring devices.

#### Care as Usual

Participants randomly assigned to the care-as-usual group will receive standard care without any restrictions and will be allowed to seek additional care or assistance for lifestyle changes if needed. Participants in the control group will be asked to fill in questionnaires and undergo the same assessments as the intervention group.

### Statistical Analysis

For descriptive statistics, categorical variables will be compared with the *χ*^2^ test (Fisher exact test when appropriate). Continuous variables will be compared using independent sample *t* test or a nonparametric test where appropriate.

To address the main hypothesis, repeated measures ANOVA using generalized linear mixed modeling analysis will be used to investigate the effect of the intervention on changes from baseline to 6 months for the 2 primary end points (Coropredict score and a 6-minute walk test) using a 2 (intervention vs control)×2 (baseline to 6 months) design, accounting for potential baseline differences and correlations between baseline and 6 months assessments. Data for the primary outcomes will be analyzed according to the intention-to-treat principle.

For the secondary outcome measures, a similar approach will be used. To explore long-term effects at 12 months, 2 (group)×3 (repeated measure) mixed models will be used. In addition, univariable and multivariable regression analyses (both linear and logistic regression) will be used to examine cross-sectional and longitudinal associations between variables, with the type of regression analysis depending on the distribution characteristics of the variables.

Exploratory analyses will also include completer analyses and evaluation of trial adherence as related to the outcome measures. As this study includes multiple secondary outcome measures without correction for statistical type 1 error, the obtained results will be interpreted with caution and will primarily provide estimates of effect sizes related to the TIMELY intervention. Data will be analyzed using SPSS statistics (IBM Corp) [48] or equivalent.

## Results

**Figure 4:**
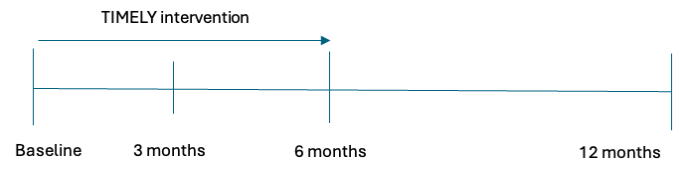
A schematic representation of the TIMELY trial.

A total of 809 patients were approached for participation. A subgroup (n=451) was excluded for not meeting the inclusion criteria or declined participation for various reasons (eg, travel burden to the hospital or perceiving the intervention as too demanding). Finally, a total of 358 patients were randomly assigned to either the intervention (n=180, 50.1%) or the care-as-usual (n=178, 49.7%) condition. In Figure 4, a schematic representation of the trial is provided, indicating the different stages of assessments. As of January 2025, all patients have completed the 6-month follow-up. The 12-month follow-up is currently underway and is expected to be completed by May 2025. Following data cleaning and analysis, results are anticipated by September 2025.

## Discussion

### Anticipated Findings

Lifestyle behaviors are a recognized risk factor and account for a significant proportion of the disease burden among people with CAD [2,21-23]. Eliminating or attenuating of these behaviors is warranted, as up to 80% of cardiovascular events can be prevented by adopting a healthy lifestyle [49,50]. However, changing and maintaining healthy behaviors is challenging, and only a subgroup of people tends to adhere to the guideline recommendations and succeed in long-term behavior change [51]. CR programs (provided online or face-to-face) are successful in changing health behaviors; however, maintaining the gains accomplished during the initial phases of CR remains challenging and requires additional support [26]. To maintain the benefits of CR, eHealth has been identified as a potentially effective approach. However, there are still gaps in the evidence supporting its use [52]. This mode of delivery provides personalized yet scalable care to those who need it. Hence, the TIMELY project aims to evaluate the effectiveness of the described patient-centered eHealth-based maintenance program, composed of an integrative care platform and an app-based, long-term health behavior change program. It is expected that supporting patients with CAD using technological innovations and applying patient-tailored and behavior-specific change techniques or domain-specific BCTs may result in significant and relevant improvements compared to care as usual. The TIMELY project goes beyond the current state-of-the-art by (1) using a highly personalized approach supported by AI technology; (2) providing a fully eHealth supported program; (3) including effective BCTs per target behavior; (4) focusing on both mental and physical factors; and (5) including the perspectives of all relevant stakeholders. In addition, evaluating cost-effectiveness is crucial for determining the economic value of the TIMELY project’s eHealth-based maintenance program. Integrating a cost-effectiveness analysis will measure not only the costs associated with implementing and using the integrative care platform and the app but also the economic benefits derived from improved health and reduced future events. This analysis will help justify the investment in these technologies by demonstrating that the proposed solutions are not only effective in terms of health outcomes but also economically efficient.

### Limitations

Given the involvement of 3 different recruitment centers with varying phase-2 CR procedures (inpatient vs outpatient CR), patient enrollment procedures might be slightly different between centers. While these differences are not expected to affect the results of the study, it is important to note that patients may be approached at different time points during or following their center-based CR. Furthermore, participants will receive several monitoring devices and extensive instructions on navigating the TIMELY app. This could be perceived by some patients as overwhelming and perhaps result in refusal or early termination of participation. Finally, as the TIMELY intervention will provide personalized advice based on incoming patient data, the intervention may be more effective for those providing data frequently.

The results of the TIMELY intervention will provide insights into the effectiveness of a fully web-based, personalized program to support long-term cardiac healthy behavior and reduce cardiac risk. The results will also provide further understanding of relevant factors contributing to successful health behavior change over time and those that help sustain these behaviors. In addition, the TIMELY project can provide new insights into which BCTs are associated with successful behavior change, helping to enhance the effectiveness of future interventions.

## Appendix

Multimedia Appendix 1 Biological specimens.

## Abbreviations

AI: artificial intelligence
BCT: behavior change technique
BP: blood pressure
CAD: coronary artery disease
CR: cardiac rehabilitation
DSS: decision support system
ECG: electrocardiogram
EP: exercise prescription
PROM: patient-reported outcome measure

## Data Availability

At the latest, by the end of the project, the data, associated metadata, documentation, and syntax codes will be deposited in a repository suitable for long-term storage or potential publishing. One of the repositories that the consortium is considering is Figshare, the repository that also serves as Universiteit van Amsterdam (timely coordinator) institutional repository. Figshare allows safely storing, controlled sharing, and publication of research data and complies with the Findable, Accessible, Interoperable, Reusable (FAIR) data principles.
