# Supplementary material for: Patient-Centered Risk Prediction, Prevention, and Intervention Platform (TIMELY) to Support the Continuum of Care in Coronary Artery Disease Using eHealth and Artificial Intelligence: Protocol for a Randomized Controlled Trial
Source: JMIR Res Protoc. 2025 Aug 14;14:e66283. doi: 10.2196/66283 (PMC12395110; doi:10.2196/66283)
Supplement: Multimedia Appendix 1 [file resprot_v14i1e66283_app1.docx]

APPENDIX 1: Biological Specimens

Blood samples will be collected at each of the study sties at three time points: baseline, six months, and 12 months. At each collection, up to 45 mL of blood will be drawn from an easily accessible vein in the arm. The blood will be collected into EDTA-vacutainer tubes and serum-vacutainer tubes.

From the EDTA-vacutainer tubes, whole blood and plasma will be processed according to the study protocol and divided into four aliquots of 1.5 ml each. Serum obtained from the serum-vacutainers will be similarly divided into 4 aliquots. An additional EDTA-vacutainer will be used to perform assays for HbA1c levels, which will be analyzed at the local hospital laboratories. All aliquots of whole blood, plasma, and serum will be stored at -80 C at the Hospital until shipment on dry ice to the core laboratory at Graz University Medical Center.

For long-term storage, the remaining blood samples will be stored using the participant’s study ID in an access-restricted are at the local research sites for a maximum period of fifteen years after study completion. This is done for follow-up determination of further blood parameters relevant to the development of risk factors for cardiovascular diseases. The reason for retaining blood samples in addition to those that are sent to the core laboratory is that scientific research is constantly evolving, and thus currently unknown blood-based biological risk factors may be discovered, which will be relevant and meaningful for this study. There will be no testing for genetic parameters (targeted examinations of individual genes or gene segments; examinations of the complete genome) based on the samples collected in this project. Any residual material will be destroyed after 15 years.

As part of the informed consent process, patients are informed about the blood collection procedures, potential discomfort (e.g. additional venipuncture), and potential risks, such as hematoma or lightheadedness.

CoroPredict score: The primary biomedical outcome measure of the TIMELY study is the change in the CoroPredict score from baseline to 6 months. The CoroPredict score is a composite index of blood-based biomarkers of cardiovascular risk factors. The biomarkers will be assessed at baseline and 6 months (for the primary outcome assessment), and also at 12 months (to explore long-term effects of the intervention). The Coropredict score assesses cardiovascular risk using a combination of laboratory-based parameters (including HbA1c, NTpro BNP, hs troponin I, cystatin C, hs C-reactive protein) and demographic information (age, sex, smoking status ).
